# Disrupted thalamic resting-state functional networks in schizophrenia

**DOI:** 10.3389/fnbeh.2015.00045

**Published:** 2015-02-25

**Authors:** Hsiao-Lan Sharon Wang, Chi-Lun Rau, Yu-Mei Li, Ya-Ping Chen, Rongjun Yu

**Affiliations:** ^1^Department of Special Education, National Taiwan Normal UniversityTaipei, Taiwan; ^2^Department of Physical Medicine and Rehabilitation, Shuang-Ho Hospital, Taipei Medical UniversityTaipei, Taiwan; ^3^Department of Psychology, National University of SingaporeSingapore, Singapore; ^4^Center for Life Sciences, Singapore Institute for Neurotechnology (SINAPSE), National University of SingaporeSingapore, Singapore

**Keywords:** thalamus, resting-state, schizophrenia, functional connectivity, fMRI

## Abstract

The thalamus plays a key role in filtering or gating information and has extensive interconnectivity with other brain regions. Recent studies provide evidence of thalamus abnormality in schizophrenia, but the resting functional networks of the thalamus in schizophrenia is still unclear. We characterize the thalamic resting-state networks (RSNs) in 72 patients with schizophrenia and 73 healthy controls, using a standard seed-based whole-brain correlation. In comparison with controls, patients exhibited enhance thalamic connectivity with bilateral precentral gyrus, dorsal medial frontal gyrus, middle occipital gyrus, and lingual gyrus. Reduced thalamic connectivity in schizophrenia was found in bilateral superior frontal gyrus, anterior cingualte cortex, inferior parietal lobe, and cerebellum. Our findings question the “disconnectivity model” of schizophrenia by showing the over-connected thalamic network during resting state in schizophrenia and highlight the thalamus as a key hub in the schizophrenic network abnormality.

## Introduction

Schizophrenia, a significant mental illness of unclear etiology, seems to affect nearly every brain structure and many brain functions. An accumulating body of evidence so far has suggested that the thalamus, a nuclear complex with multiple connections to different parts of the brain regions may be abnormal in patients with schizophrenia (Jones, 1997). It is therefore believed that faulty connections between the thalamus and other cortical regions may be related to the wide diversity of behavioral and cognitive characteristics appeared in those patients (Mingoia et al., 2012). Although many *in-vivo* neuroimaging studies have implicated the thalamus in the pathophysiology of schizophrenia, limitations of constant methods of evaluating thalamic structure and function have prevented researchers from clearly understanding its role and findings have been largely inconsistent (Woodward et al., 2012).

While cortical organization considerations in prior research received much attention, an equally large literature has focused on thalamus and its role. Particularly, the thalamic dysfunction hypothesis has been a major recent model for schizophrenia and accounts for the onset and progression of this disease. Aberrant interconnectivity of the thalamus with other brain regions is presumed to play a central role in mental functions that are focal points of schizophrenia such as consciousness, perception, and the integration of thoughts (Bob and Mashour, 2011; Angelopoulos et al., 2014). In other words, a misconnectivity or disconnectivity syndrome would affect the organization of neurons in the brain creating a problem in neural connection (Schmitt et al., 2011). This assumption has been examined with functional magnetic resonance imaging (fMRI) and EEG (electroencephalography) data. Theoretically, brain regions, which have spontaneous synchronized neuronal activity are “functionally” connected (those regions exhibited synchronized activity because of the causal effect of one brain region had on another). EEG studies have revealed that patients with schizophrenia have decreased synchronization of neuronal oscillations, especially beta and gamma band activity (Uhlhaas and Singer, 2010). Studies using fMRI and the blood oxygenated level dependent (BOLD) contrast showed that patients with schizophrenia have decreased task and resting state functional connectivity (FC) of different brain regions (Whalley et al., 2005) and alterations of the default mode network (DMN; Mingoia et al., 2012). Although most researchers agree that cerebral connectivity may be altered in schizophrenia, still we cannot agree on a consistent set of alterations that can be used to characterize the mental problem.

To better understand whether connectivity differences between the thalamus and cortical regions may have contributed to schizophrenics’ clinical symptoms, we investigated a conventional metric of resting state connectivity. Typically, brain functional resting-state networks (RSNs) characterize a corresponding set of significantly coherent brain areas with respect to low-frequency BOLD signals during resting state (Zhang et al., 2008; Welsh et al., 2010). The correlations between sensorimotor cortical areas and DMNs during the resting state have been substantially mentioned in previous schizophrenia research (Tang et al., 2011; Klingner et al., 2014). However, the resting functional networks of deep gray matter structures, such as the thalamus, have been seldom studied with resting-state fMRI. Nevertheless, findings so far seem consistent to suggest a qualitative difference between patients’ with schizophrenia and healthy controls in the thalamus related FC. Previous data showed a typical pattern of thalamic functional RSNs in healthy individuals that may involve several cortical regions (Tang et al., 2011). For example, motor and somatosensory areas were correlated strongly with ventral lateral and ventral posterior-lateral portions of the thalamus. Using ROI (region of interest) based approach, the prefrontal cortex was found functionally connected to anterior and dorsomedial regions of the thalamus, whereas the temporal lobe and occipital cortex were found to be correlated with posterior medial and lateral areas of the thalamus that appeared consistent with the medial geniculate nucleus and the lateral geniculate nucleus, respectively (Zhang et al., 2010). Studies also indicated that patients with schizophrenia suffered from disruption in their thalamic RSNs, manifesting both hyper- and hypo-regulation of thalamocortical FC. A recent study by Woodward et al. (2012) suggested a significant group difference in the prefrontal-thalamic connectivity and motor/somatosensory-thalamic connectivity (Woodward et al., 2012). Group comparisons revealed significantly reduced prefrontal-thalamic connectivity and increased motor/somatosensory-thalamic connectivity in schizophrenia, after controlling the local gray matter content within the thalamus and their antipsychotic medication dosage.

Built upon the literature, our purpose in this study was to understand the neural correlates of the thalamus by using resting-state fMRI and to investigate whether thalamic RSNs are different in patients with schizophrenia. Advanced from the prior research, here we surveyed the FC/coupling between a seed region (thalamus) and other cortical and subcortical areas in the whole brain during the resting condition. The analysis of intrinsic functional architectural changes by using resting-state fMRI in schizophrenia may help us to identify the pathophysiological mechanisms of disease, to improve the clinical observation and diagnosis and suggest better interventions.

## Materials and methods

### Participants

Seventy two patients with schizophrenia (58 males, 14 females, see Table 1), according to DSM-IV diagnostic criteria, were studies from the dataset released from the Center for Biomedical Research Excellence (COBRE), University of New Mexico.^1^ Patients with the presence of DSM-IV Axis I diagnoses of other disorders such as depression, a history of any substance dependence, or a history of clinically significant head trauma were excluded from the analysis. The treatment details of schizophrenia patients were listed in Table 2. Meanwhile, 74 age-compatible healthy controls were recruited (two were dis-enrolled). All of the control participants were free of the DSM-IV diagnoses of schizophrenia and other DSM-IV Axis I diagnoses of mental disorders. None of them had neurological diseases, a history of any substance dependence, or a history of clinically significant head trauma.

**Table 1:** **Sample Demographics**.

Measure	Schizo (*n* = 72)		HC (*n* = 74)		Statistics
	Mean	SD	Mean	SD	*P*
Age (year)	38.17	13.89	35.82	11.58	0.27
Gender					0.15
Male	58		51
Female	14		23
Handness					< 0.01^b^
Right	60		71
Left	10		1
Both	2		2
IQ	(*n* = 68)		(*n* = 67)
Verbal	97.88	16.73	106.79	11.16	< 0.01^b^
Performance	102.68	16.64	114.03	12.32	< 0.01^b^
Sum	99.59	16.86	108.33	11.83	< 0.01^b^
Education (year)	12.99	1.84	13.52	1.75	< 0.01^b^
Illness duration (year) (*n* = 71)	16.03	12.41
PANSS
Positive scale (*n* = 72)	14.96	4.83
Negative scale (*n* = 72)	14.53	4.83
General (*n* = 72)	29.22	8.34

*Note: Demographic information for the patient sample and control sample. Mean and standard deviation are provided for the continuous variable (e.g., age). Schizo = schizophrenic patients. HC = healthy controls. ^a^Fisher’s exact test, *p* < 0.01, ^b^Student’s t test, p < 0.01*.

**Table 2:** **Treatment details of schizophrenia patients**.

Treatment	Case number
Risperdal/Risperidone	15
Seroquel/Quetiapine	12
Aripiprazole/Abilify	11
Clozapine/Clozaril	11
Zyprexa/Olanzapine	11
Risperdal Consta/Risperidone Microspheres	11
Ativan/Razepam	8
Sertraline/Zoloft	8
Geodon/Ziprasidone	7
Benzotropine/Cogentin	6
Propranolol/Inderal	6
Holdol Dec/Haloperidol decanoate	6
Trazaone/Desyrel	4
Clonazepam/Klonopin	3
Buproprion/Wellbutrin	3
Citalopram/Celexa	3
Fluoxetine/Prozac	3
Zolpidem/Ambien	2
Divalproex/Depakote	2
Diazepam/Valium/Diastat/Diastat	1
Zaleplon/Sonata	1
Lexapro/Escitalopram	1
Fluvoxamine/Luvox	1
Mirtazapine/Remeron	1
Effexor/Venlafaxine	1
Tegretol/Carbamazepine	1
Permatil/Fluphenazine	1
Haloperidol/Haldol	1
Trilafon/Perphenazine	1
Thiothixene/Navane	1

### MRI data acquisition

All subjects underwent structural and functional MRI scans in a single session using a 3 T Siemens Trio scanner with foam padding/paper tape to restrict head motion. All images were acquired parallel to anterior-commissure-posterior-commissure line with an auto-align technique.

For the reference image of anatomy and ROI analyses, a five-echo multiecho magnetization-prepared rapid gradient echo (MEMPR) sequence was used to acquire a whole brain high-resolution T1-weighted MR image in a coronal view [TE (echo times) = 1.64, 3.5, 5.36, 7.22, 9.08 ms, TR (repetition time) = 2.53 s, inversion time = 1.2 s, flip angle = 7°, number of excitations = 1, slice thickness = 1 mm, FOV (field of view) = 256 mm, resolution = 256 × 256]. The total scan time was 6.325 min and 150 time points were collected. Echo-planar images (336 images across three runs) were collected using a conventional single-shot, gradient-echo echoplanar pulse sequence [TR = 2,000 ms; TE = 29 ms; flip angle = 75°; FOV = 240 mm; matrix size = 64 × 64; 33 slices; voxel size = 3.75 × 3.75 × 4.55 mm^3^]. The first image of each run was eliminated to account for T1 equilibrium effects (Mayer et al., 2013).

The resting state fMRI was performed with single-shot full k-space echo-planar imaging (EPI) with ramp sampling correction using the intercomissural line (AC-PC) as a reference. The fMRI acquisition parameters were as follows: TR/TE = 2000 ms/29 ms, FOV = 256 mm × 256 mm, matrix = 64 × 64, 32 slices, voxel size: 3 × 3 × 4 mm^3^ (see http://coins.mrn.org/dx for more details).

### Image processing

The first 5 volumes were not analyzed to allow for signal equilibration effects. The fMRI data were then preprocessed using SPM8 software (available at: http://www.fil.ion.ucl.ac.uk/spm) implemented in a MATLAB suite (Mathworks, Inc, Natick, Massachusetts). Images were realigned to correct for motion, corrected for errors in slice timing, spatially transformed to standard stereotaxic space (based on the Montreal Neurologic Institute coordinate system), and smoothed with a 6 mm full-width half-maximum gaussian kernel. Data were then bandpass filtered from 0.01 to 0.08 Hz, to remove low frequency noise (including slow scanner drifts) and influences of higher frequencies reflecting cardiac and respiratory signals (Cordes et al., 2001).

Functional connectivity analysis was carried out by applying a seed-region approach using the left and right thalamus as defined in the automated anatomical labeling atlas (AAL; Tzourio-Mazoyer et al., 2002). For each ROI, individual participant analyses were carried out using the General Linear Model (GLM) with the time series for the ROI, as well as for the nuisance covariates (white matter, cerebrospinal fluid, and six motion parameters) as predictors. These nuisance signals are typically adjusted for in resting-state FC studies (Yu et al., 2014a,b), in order to remove the influence of global signal fluctuations of nonneuronal origin (e.g., physiological artifacts associated with variables such as cardiac and respiratory cycles, CSF motion, and scanner drift) (Fox and Raichle, 2007).

To address head motion concerns in resting-state fMRI analyses, we calculated the voxel-specific mean framewise displacement (FD) for accounting head motion at group-level analysis (Power et al., 2012, 2013, 2014; Van Dijk et al., 2012). FD measure indexes the movement of the head from one volume to the next and is calculated as the sum of the absolute values of the differentiated realignment estimates (by backward differences) at every time point (Power et al., 2012). Then, we repeated the above analyses after removing frames with FD > 0.5 mm (“scrubbing”). One time point before “bad” time points and one time points after “bad” time points were deleted. Eleven participants in the schizophrenia group and two in the control group were excluded because that more than 70% of the volumes have FD > 0.5 mm in these subjects. In the remaining 60 schizophrenia, 22 ± 19 (mean ± SD) percent of time points were removed, leaving 113 ± 27 time points. There was no significant difference between the excluded schizophrenia subjects and the included schizophrenia subjects in symptoms (i.e., PNASS and illness duration), *p* values >0.2, indicating that greater movement in those subjects were not due to worse symptoms. The mean FD in the schizophrenia group was significant larger than that in the control group, *t* = 3.12, *p* < 0.005. Thus, the FD value for each subject was added as covariate of no interest in all fMRI analysis.

### Statistical analysis

Contrast images were generated for each subject by estimating the regression coefficient between all brain voxels and each seed’s time series, respectively. The resultant *z*-transformed *β*-value maps were then included in group (second-level) random effects analyses, adopting a 2 × 2 mixed design, factorial model (group [control, patient] by hemisphere [right seed, left seed]). Moreover, adding age, sex, and education level as additional covariate of no interest did not change the results. We also used regression analyses to examine whether clinical scores (e.g., PANSS and IQ scores) were related to thalamic FC, when considering the schizophrenia participants alone. The threshold was *P* < 0.05, family wise error (FWE) correction for multiple comparisons at the cluster level. The threshold of *P* < 0.001 uncorrected was used as cluster forming threshold. The cluster level correction method has better sensitivity than voxel-level correction at the cost of poor localization power. Maps are displayed at the same threshold (corrected at cluster level). Images are in radiologic format with subject left on image right. All coordinates are reported in MNI coordinates by SPM.

## Results

Table 1 represents the sample demographics. For the schizophrenia group, one subject was removed because only 67 time pointed were collected for this subject. As shown in the Table 1, the average age of our 72 schizophrenia patients was 38.17 ± 13.89 years. The PANSS scales were reported in the patients’ group. In contrast, the average age of control subjects was 35.82 ± 11.58 years. There was a significant group difference in their personal educational attainment, *p* = 0.008. This was expected, as patients’ psychiatric disorders often hold back learning achievement in other schizophrenia samples (Guo et al., 2013; Yu et al., 2014a). Within these participants, ten in the schizophrenia group and only one in the control group were left-handed. Demographic data also suggests our sample groups did not significantly differ in gender distribution (*χ*^2^ = 2.033, *p* = 0.154) or average age (*t* test, *p* = 0.270).

**Figure 1:**
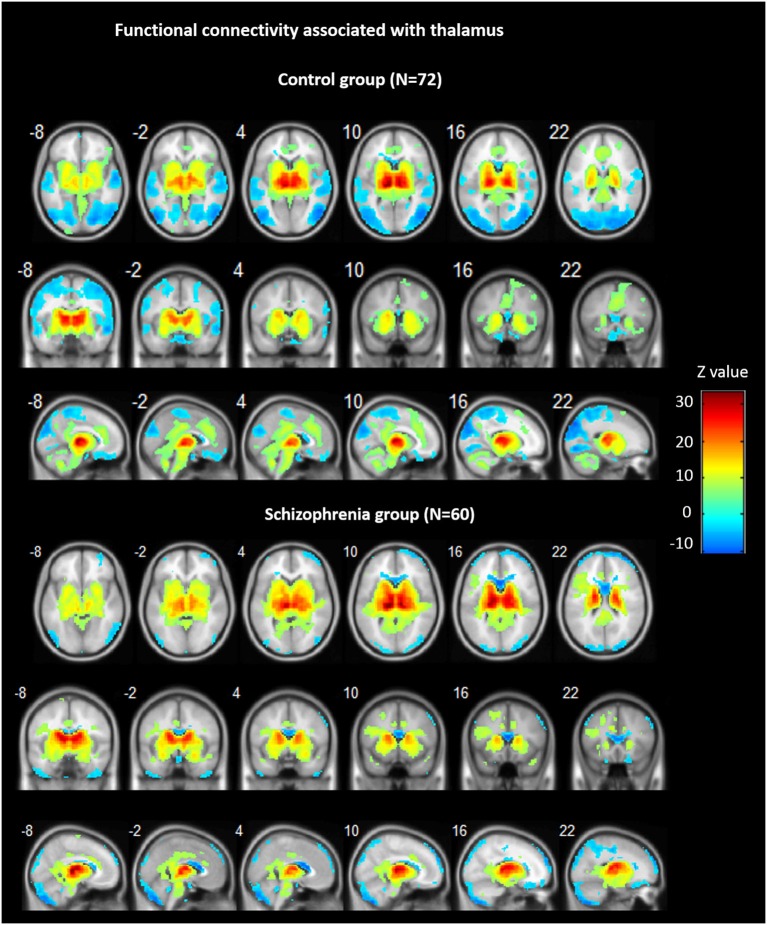
**Functional connectivity (FC) associated with the thalamus in schizophrenia and in controls**. Hot color represents positive FC with thalamus, whereas blue cold color represents negative FC. Images are in radiologic format with subject left on image right.

Across groups, FC analyses for the thalamus seeds revealed significant positive correlations with ACC/dorsal medial prefrontal cortex (PFC), bilateral insula, bilateral striatum, midbrain, and cerebellum, as well as negative correlations bilaterally with ventral medial frontal gyrus, precentral gyrus, cuneus, precuneus, and lingual gyrus (see Figure 1).

**Figure 2:**
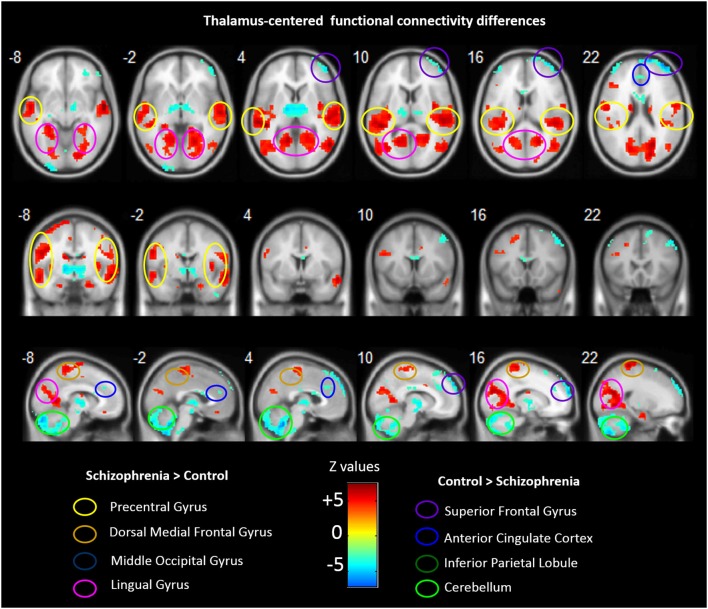
**Abnormal FC associated with the thalamus in schizophrenia**. Hot color represents higher FC with thalamus in schizophrenia, whereas blue cold color represents lower FC in schizophrenia. Images are in radiologic format with subject left on image right.

Group analyses revealed that schizophrenia patients exhibited reduced thalamus-based FC with bilateral superior frontal gyrus, anterior cingualte cortex, inferior parietal lobe, and cerebellum. Patients also showed increased thalamus-based FC with bilateral precentral gyrus, dorsal medial frontal gyrus, middle occipital gyrus, and lingual gyrus (see Table 3; Figure 2). There was no significant interaction between group and hemisphere.

**Table 3:** **Brain regions showing differences in the thalamus-based functional connectivity between controls and patients with schizohrenia**.

Brain Regions	Voxels	Z-scores	MNI Coordinates		
			*X*	*Y*	*Z*
Control vs.Schizophrenia
Thalamus	176	5.32	12	−9	3
Thalamus			−12	−5	0
Midbrain			6	−15	−15
Superior Frontal Gyrus	400	6.53	−33	54	30
Inferior Parietal Lobule	122	5.32	−60	−48	42
Anterior Cingualte Cortex	65	3.80	−12	33	27
Cerebellum	1737	6.46	33	−63	−54
Cerebellum	442	5.24	−30	−60	−39
Schizophrenia vs. Control
L Superior Temporal Gyrus	3394	6.54	−60	−6	−9
Precentral Gyrus		6.25	−42	−21	66
Precentral Gyrus		5.83	35	−27	69
Posterior insula		5.27	−55	−18	0
Dorsal medial PFG		5.05	3	−30	63
R Superior Temporal Gyrus	728	6.39	−21	−50	−3
Posterior insula		6.03	45	−36	6
Lingual Gyrus	1738	4.97	54	−9	−12
Middle Occipital Gyrus	231	4.62	36	−72	9
Middle Occipital Gyrus	283	5.79	−54	−63	3

*All values P < 0.05 FWE-corr at cluster-level after t maps thresholded at P < 0.001 uncorrected*.

Regression analysis found no significant thalamic FC with clinical variables.

## Discussion

In this study, we reexamined the fMRI data of a large group of schizophrenia patients obtained from the COBRE database, University of New Mexico regarding thalamic FC during resting state. Thalamus-based FC was established across all brain regions in all subjects. However, we found significant regional differences in the connectivity patterns of patients. The results partly supported the findings by Woodward et al. (2012) and displayed both over and under connectivity in the thalamic network during resting state (Woodward et al., 2012), supporting the view that schizophrenia is characterized by a mixture of hypo- and hyper-connectivity (Pettersson-Yeo et al., 2011).

In our study, schizophrenia patients exhibited increased thalamic FC in several brain areas, including bilateral precentral gyrus, dorsal medial frontal gyrus, middle occipital gyrus, and lingual gyrus. Our findings were not altogether consistent with those of previous studies. Similar to many prior studies, thalamic hyper-connectivity was detected in motor and visual cortices. These hyper-connectivity findings challenge the disconnectivity/hypoconnectivity hypothesis of schizophrenia which states that too many synapses are eliminated in development, specifically during adolescence, in schizophrenia (Beaumont and Dimond, 1973; Friston and Frith, 1995; Friston, 1999). Our results are in favour of the misconnectivity hypothesis that the regulation of synaptic strength in the connections between cortical neurons is altered in schizophrenia and may be manifested as both hyper- and hypo-connectivity (Crow, 1998; Innocenti et al., 2003). Using voxel-based morphometry and Granger causality analysis, a recent study demonstrated that the causal connectivity of the integrated prefrontalthalamic (limbic)-cerebellar (sensorimotor) circuit was partly affected by structural deficits in first-episode, drug naive schizophrenia (Guo et al., 2015), although no age thalamic connectivity was found in that study. A recent study further examined whether and how FC patterns during rest change with task engagement in schizophrenia (Cetin et al., 2014). Using independent component analysis (ICA) combined with experimental tasks with different levels of sensori-motor processing, this study found higher functional network connectivity between thalamus and multiple sensorimotor regions across tasks, highlighting the importance of the thalamus as a gateway to sensory input (Cetin et al., 2014). This study, using a different data analysis approach (i.e., ICA), provides convergent evidence that thalamic FC with other sensorimotor areas is exaggerated in schizophrenia and further shows that this pattern persists even during sensory load tasks. Taken together, these findings highlighted a key role of thalamus in the neurodevelopment of FC network in schizophrenia.

Hyper-connectivity was observed in dorsal medial frontal gyrus, while hypo-connectivity has been identified in PFC (Woodward et al., 2012). We suspect that the hyper-connectivity between thalamus and dorsal medial prefrontal gyrus (dmPFG) might be related to the impaired decision making found in schizophrenics. Patients with schizophrenia are characterized by making decisions based on less information, which results in fast and incorrect decisions. This behavior is referred to as jumping to conclusions bias (Averbeck et al., 2011; Krug et al., 2014). The underlying mechanism might be linked with the crucial thalamic function of filtering and gating information (Andreasen, 1997) and the function of medial frontal gyrus in high-level executive functions and decision-related processes (Mansouri et al., 2009). Talati and Hirsch (2005) noted that medial frontal gyrus was associated with information collection of perceptual go/no-go decision making. Specifically, lower-level processing biases were determined and left hemisphere of the medial frontal gyrus processes perceptual decisions depending on temporal and object information. The right hemisphere of the medial frontal gyrus conducted analogous decisions based on spatial information. As a result, thalamus-dorsal medial prefrontal gyrus hyper-connectivity might readily activate this pathway and exert influences on the decision making of schizophrenia patients.

In our results, thalamic hyper-connectivity was also found in lingual gyrus in schizophrenics. Lingual gyrus and middle occipital gyrus are parts of the occipital lobe, where visual cortex locates. Starting in the 1950s, the symptom of visual perception disturbances has been widely reported in schizophrenia patients (Silverstein and Keane, 2011). This might provide an illustration that schizophrenics typically performed worse in visual contrast (Dakin et al., 2005; Yang et al., 2013) and visual motion and orientation tasks (Yang et al., 2013). In addition, the lingual gyrus contributes to visuomotor processing and plays a key role in generating or recalling dreams during sleep (Bischof and Bassetti, 2004). Thus, this alternation of connectivity between the lingual gyrus and the thalamus might be related to an underlying sleep disturbance in schizophrenic patients. Perhaps the increased connectivity between the lingual gyrus and the thalamus caused severe sleep latency and sleep disorders in schizophrenia patients.

Consistent with several studies reported decreased thalamic connectivity in PFC (Woodward et al., 2012), our data indicated thalamic hypo-connectivity in subregions of the PFC, including bilateral superior frontal gyrus. In schizophrenia patients, decreased connectivity between PFC and working memory related regions such as thalamus was found to be linked to deficits in the central executive component of working memory (Barch and Csernansky, 2007; Barch and Ceaser, 2012). Our finding provided more detailed locations of brain areas affected by schizophrenia. In an fMRI study, Tully et al. (2014) reported that thinner cortex was found in a region in the superior frontal gyrus (Brodmann area 10) of schizophrenia patients and was associated with declined role and real-world functioning. Thinner cortex and poor function in the superior frontal cortex might result from hypo-connectivity between the area and thalamus. Subsequently, thalamic hypo-connectivity caused declined role and real-world functioning in schizophrenics.

In addition, our results showed thalamic hypo-connectivity in anterior cingulate cortex, inferior parietal lobs, and cerebellum. In an electrophysiological study, Mulert et al. (2001) suggested that anterior cingulate cortex activity of schizophrenics was reduced as indicated by the attenuated N1 potential (60–150 ms). Yan et al. (2012) presumed that decreased resting-state connectivity in anterior cingulate cortex was crucial in schizophrenia symptom expression and was correlated with Stroop colour-word test performance and disease severity. Stroop colour-word test was used to test executive control function. The study therefore concluded that deficits in executive control function were related to schizophrenics’ abnormal functional and structural connectivity in anterior cingulate cortex. Our results provided further evidence for the implication of anterior cingulated cortex in schizophrenia.

On the other hand, studies regarding a DMN may shed light on the thalamic hypo-connectivity in inferior parietal lobes. The DMN comprised medial prefrontal cortex, posterior cingulate cortex/precuneus, inferior parietal lobule, and lateral temporal cortex. In passive setting and when direct attention was not required, the DMN was activated (Raichle et al., 2001; Buckner et al., 2008). Previous studies demonstrated diverse DMN connectivity patterns (Zhou et al., 2007; Whitfield-Gabrieli et al., 2009; Ongür et al., 2010; Rotarska-Jagiela et al., 2010; Jang et al., 2011; Calhoun et al., 2012; Mingoia et al., 2012), which might explain the heterogeneous symptoms of this disease. In our current data, schizophrenia patients had a different pattern of DMN, where thalamus and inferior parietal lobule displayed lower FC in schizophrenics than that did in normal controls. Our findings also indicated a reduced FC in medial prefrontal cortex/ACC and supported the assumption of decreased connectivity in DMN found in schizophrenics (Rotarska-Jagiela et al., 2010; Camchong et al., 2011; Jang et al., 2011). This atypical intrinsic connectivity is thought to associate with the difficulties in self-referential, introspective processing as well as theory of mind which often seen in schizophrenia patients (Zhou et al., 2007; Camchong et al., 2011; Liu et al., 2012). Interestingly, in some other references, first-degree relatives of schizophrenia patients, including unaffected siblings, have been reported to reveal similar atypical intrinsic connectivity that is observed in patients, although patients tend to exhibit more severe and widespread aberrances (van Buuren et al., 2012; Chang et al., 2014; Guo et al., 2014). It seems that the degree of this intrinsic connectivity dysfunction might be related to the risk of developing schizophrenia the illness manifestation. It also worth noting that the DMN may not be a single unit but is composed of substructures with substantially different connectivity patterns. Schizophrenia might be associated with abnormalities in certain subcomponents of the DMN but not others (Calhoun et al., 2012).

Consistent with a prior study that employed the same dataset provided by the COBRE, our results also showed decreased cerebellar-thalamic FC (Wang et al., 2014). Several resting-state fMRI studies as well have observed the same results of thalamic hypo-connectivity in cerebellum (Collin et al., 2011; Chen et al., 2013). In a diffusion tensor tractography study, schizophrenia patients had a declined fractional anisotropy in fiber tracks between cerebellum and thalamus (Magnotta et al., 2008). Previous literature has reported that the cortico-cerebellar-thalamic-cortical circuit (CCTCC) dysfunction might cause some symptoms of schizophrenia (Andreasen et al., 1998, 1999). Cerebellum impairment might result in the CCTCC dysfunction and subsequent schizophrenia symptoms. Other than movement synchrony, cerebellum was also responsible for cognitive and affective functions (Leiner et al., 1993; Stoodley et al., 2012). The hypo-connectivity between the cerebellum and thalamus might elucidate the neurological soft signs of schizophrenia patients because those patients also exhibited cerebellar dysfunction (Ho et al., 2004; Varambally et al., 2006).

Our study has several limitations. First, schizophrenia patients may have diverse antipsychotic medication statuses, which might affect their brain activities (Lui et al., 2010; Sambataro et al., 2010) and cognitive functions (Meltzer and McGurk, 1999). According to the literature, after receiving 6-week treatment of second-generation antipsychotic drugs, synchronized brain activity and declined integration function across brain networks were identified among schizophrenics (Meltzer and McGurk, 1999). Sambataro et al. (2010) also acknowledged that olanzapine treatment increased DMN connectivity in schizophrenics. Second, we need empirical and longitudinal data to establish the causal relationship between FC and schizophrenia symptoms. Our analysis simply reanalysed an existing dataset; therefore, lacking hypothesis-driven experiment design to determine the causal relationship between brain structures and schizophrenia symptoms. Future studies can develop longitudinal studies to ascertain the causal relationship. Finally, the current study used the entire left and right thalamus as seeds. However, it is probable that there is some gradient in connectivity within the thalamus. Future studies may use sub-regions in the thalamus as seeds to further explore the FC within different parts of thalamus.

This study re-examined a dataset with a great number of schizophrenia patients. The analytical method adopted by this study yielded results of both thalamic hyper- and hypo-connectivity in more detailed and specific brain regions. For example, we identified thalamic hyper- and hypo-connectivity in two subregions of the PFC, dorsal medial frontal gyrus and bilateral superior frontal gyrus, respectively. In conclusion, our results demonstrated a coherent finding that schizophrenics had a loosely integrated and more diverse FC across the brain. Precise locations of brain regions involved in thalamic FC not only showed a clearer brain mapping of schizophrenia patients but also provided more clues for future researchers.

## Conflict of interest statement

The authors declare that the research was conducted in the absence of any commercial or financial relationships that could be construed as a potential conflict of interest.

## Footnotes

^1^http://coins.mrn.org/dx and http://fcon_1000.projects.nitrc.org/indi/retro/cobre.html
